# Ectoparasitic fungi of *Myrmica* ants alter the success of parasitic butterflies

**DOI:** 10.1038/s41598-021-02800-3

**Published:** 2021-12-15

**Authors:** András Tartally, Norbert Szabó, Anna Ágnes Somogyi, Ferenc Báthori, Danny Haelewaters, András Mucsi, Ágnes Fürjes-Mikó, David R. Nash

**Affiliations:** 1 Department of Evolutionary Zoology and Human Biology, University of Debrecen, Egyetem tér 1, 4032 Debrecen, Hungary; 2 Juhász-Nagy Pál Doctoral School of Biology and Environmental Sciences, University of Debrecen, Egyetem tér 1, 4032 Debrecen, Hungary; 3 Department of Zoology, Hungarian Natural History Museum, Baross str. 13, 1088 Budapest, Hungary; 4 Evolutionary Ecology Research Group, Institute of Ecology and Botany, Centre for Ecological Research, Alkotmány út 2-4, Vácrátót, Hungary 2163; 5 Research Group Mycology, Department of Biology, Ghent University, K.L. Ledeganckstraat 35, 9000 Ghent, Belgium; 6 Faculty of Science, University of South Bohemia, Branišovská 31, 370 05 České Budějovice, Czech Republic; 7 Bezerédi str. 10, 5462 Cibakháza, Hungary; 8 Department of Forest Protection, University of Sopron-Forest Research Institute, Hegyalja str. 18, 3232 Mátrafüred, Hungary; 9 Centre for Social Evolution, Department of Biology, University of Copenhagen, Universitetsparken 15, 2100 Copenhagen, Denmark

**Keywords:** Conservation biology, Fungi, Entomology, Infection

## Abstract

Exploitation of organisms by multiple parasite species is common in nature, but interactions among parasites have rarely been studied. *Myrmica* ants are rich in parasites. Among others, the ectoparasitic *Rickia wasmannii* fungus and the parasitic caterpillars of myrmecophilous *Phengaris* butterflies often infect the same *Myrmica* colonies. In this study, we examined the effects of *R. wasmannii* on the adoption, long-term development, and survival of *P. alcon*. In laboratory conditions, caterpillars introduced into nests of *Myrmica scabrinodis* uninfected with *R. wasmannii* survived significantly longer compared to caterpillars introduced into infected nests. In the field, joint infection was less common than expected if both parasites exploited *M. scabrinodis* colonies independently. Pre-pupal caterpillars of *P. alcon* were somewhat larger in nests infected with *R. wasmannii* than those found in uninfected nests. Based on these results it seems that *R. wasmannii* infection of *M. scabrinodis* affects the survival and development of *P. alcon* caterpillars, suggesting competition between these two ant parasites.

## Introduction

Organisms are often exploited by multiple parasites in nature, but this is rarely highlighted in ecological studies^1–3^. From the point of view of the host, co-infecting parasites may have synergistic, additive, or antagonistic effects on fitness^1,4,5^. Co-infection may also result in fitness-changing interactions between the parasites^2,6^, as they may show contest or scramble competition for host resources^1^, or have more complex interactions^7–9^. When any of the species involved in such interactions are of conservation concern, then an understanding of the effects of multiple infections on both hosts and parasites is vital. While the importance of conserving parasites is being increasingly acknowledged^10–12^, the threats or protection offered by co-infecting parasites has largely been ignored.

Colonies of ants (Hymenoptera: Formicidae) are subject to numerous parasites, ranging from pathogens and endo- and ecto- macroparasites and parasitoids that exploit individuals, to social parasites that exploit entire colonies and their resources^13–19^. This makes ant colonies good model organisms for parasitological studies.

*Myrmica* ants are particularly rich in parasites. Their colonies often provide a home for different social parasites, including other *Myrmica* species, *Lomechusa* beetles (Coleoptera: Staphylinidae), *Platyarthrus* woodlice (Isopoda, Platyarthridae), *Cyphoderus* springtails (Entomobryomorpha: Paronellidae), larvae of *Microdon* hoverflies (Diptera: Syrphidae) and the caterpillars of *Phengaris* (= *Maculinea*) butterflies (Lepidoptera: Lycaenidae). *Myrmica* individuals can be infected by parasitoid wasps (Hymenoptera: Eucharitidae and Ichneumonidae) and flies (Diptera: Phoridae), endoparasitic nematodes (Mermithidae, Rhabditidae and Steinernematidae), ecto- and endoparasitic fungi, and various bacteria^20–23^. While there is some information on the co-infection levels of *Myrmica* nests by different species of *Phengaris* butterflies and *Microdon myrmicae*^24,25^ the consequences of these co-infections for the parasites themselves is poorly understood.

**Figure 1:**
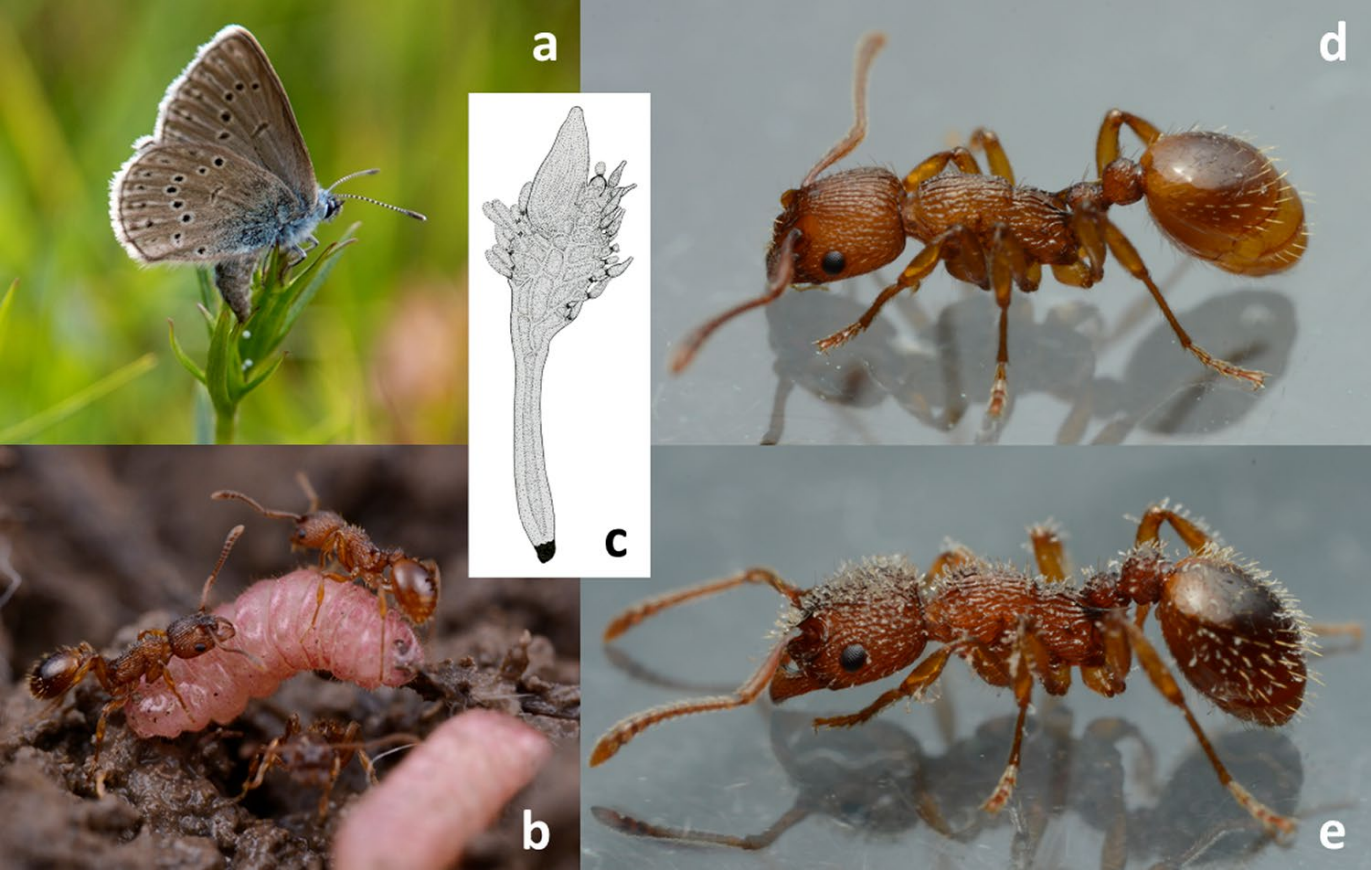
Illustrations of study organisms. (**a**) *Phengaris alcon* female laying eggs on *Gentiana pneumonanthe* flower buds. (**b**) Overwintered *P. alcon* caterpillars in a *Myrmica scabrinodis* nest. (**c**) Drawing of an adult *Rickia wasmannii* thallus. (**d**) *Myrmica scabrinodis* worker uninfected with *R. wasmannii*. (**e**) *Myrmica scabrinodis* worker with numerous *R. wasmannii* thalli on its cuticle; see also Supplementary Video S1. Photo: David R. Nash (**a**), Ádám Bakos (**b**, **d**, **e**); the drawing (**c**) is in the public domain, from the original illustrations by Roland Thaxter, courtesy of the Farlow Reference Library of Cryptogamic Botany, Harvard University.

Over the last two decades we have investigated two parasites of *Myrmica* ants in detail (Fig. 1, Supplementary Video S1), socially parasitic *Phengaris* butterflies and the ectoparasitic fungus *Rickia wasmannii* (Ascomycota: Laboulbeniales). Both affect their *Myrmica* host ants, but in different ways. *Phengaris* caterpillars have a strong negative effect on their host colonies by feeding on their brood and/or by receiving food by trophallaxis that would otherwise go to ant workers or larvae^26,27^. *Rickia wasmannii* increases the need for water of the host ants, makes them less aggressive and less bold, increases allo- and auto-grooming frequency, reduces the size of workers and the thickness of their cuticle and, in general, causes earlier death of workers, at least in the lab^14,28–32^. Despite the fact that these two organisms can co-occur within the same *Myrmica* colonies^33^, we know of only a single study addressing the effects of common infection. Csata et al.^14^ found that *M. scabrinodis* colonies infected by *R. wasmannii* adopted a higher proportion of offered larvae of *Phengaris teleius* and *P. alcon* compared to uninfected colonies, although for *P. alcon* differences were mostly due to differences in discovery of caterpillars rather than caterpillar rejection. In that study, the caterpillars had only 120 min to be discovered and transported by the ants to the nest. However, short-term adoption does not mean the integration of caterpillars into the *Myrmica* colonies and their successful development to adult butterflies^34,35^.

**Figure 2:**
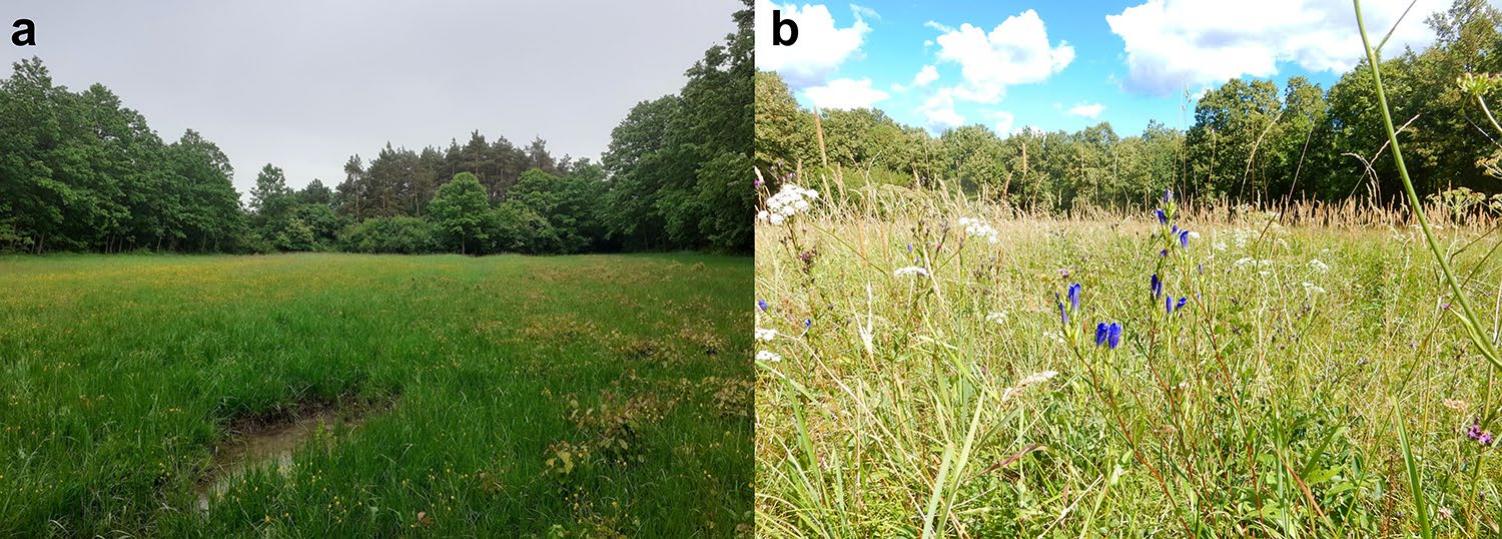
The field site at Gyilkos-rét where *Myrmica scabrinodis* nests and *Phengaris alcon* eggs and caterpillars were collected. (**a**) Early June. (**b**) Mid-August with flowering *Gentiana pneumonanthe* in the central foreground. See Fig. 5 in Csősz et al.^29^ for maps of the locality. Photo: András Tartally (**a**) and Márton József Paulin (**b**).

**Figure 3:**
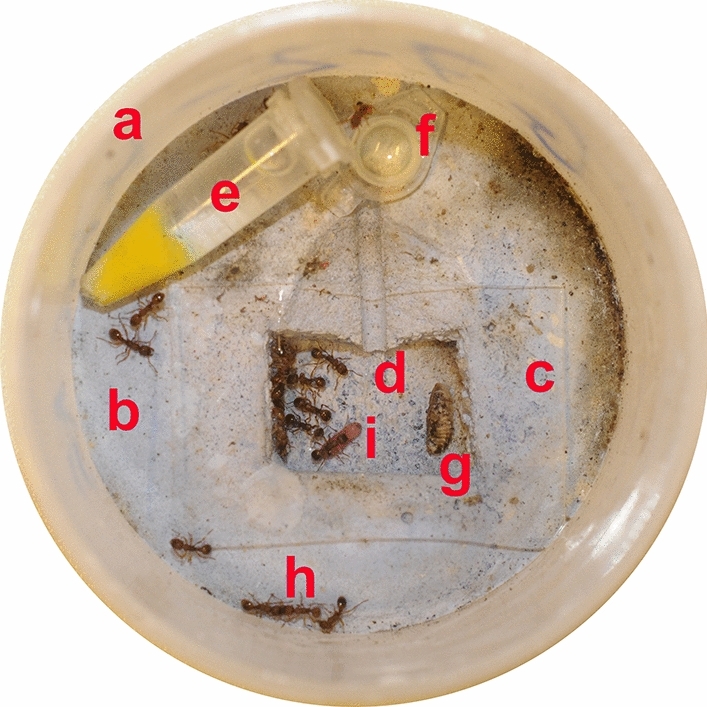
Nest design. (**a**) Plastic box. (**b**) Plaster floor. (**c**) Glass plate. (**d**) Nest chamber with entrance. (**e**) “Drinker” (a 1.5 mL Eppendorf tube with cut tip, stuffed with a piece of cloth and filled with tap water). (**f**) Honey-sugar water in a “feeder” (the cap of an 1.5 mL Eppendorf tube). (**g**) Insect food (dead cockroach). (**h**) *Myrmica scabrinodis* workers. (**i**) Young *Phengaris alcon* caterpillar with a *M. scabrinodis* worker. The diameter of the nest is 60 mm. Photo: Ferenc Báthori.

**Figure 4:**
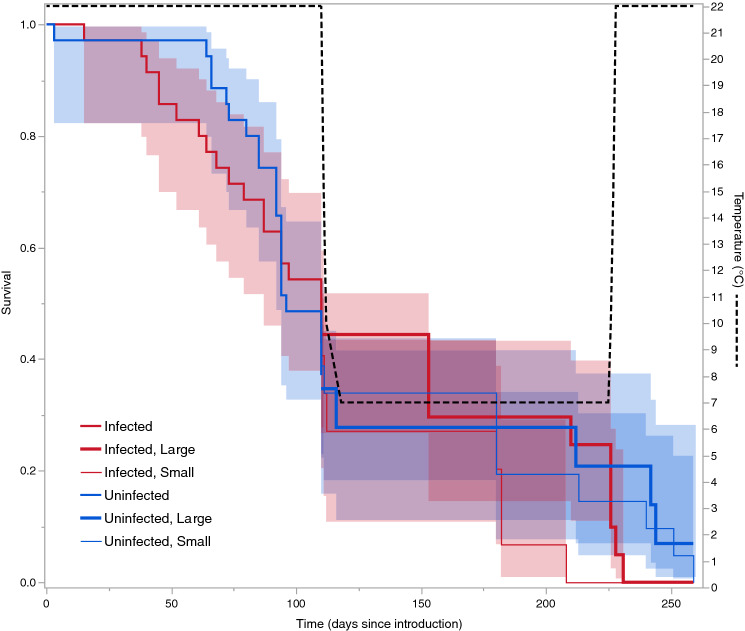
Survival of caterpillars in the mini colonies, and temperature at which they were kept. Before overwintering (drop in temperature after 110 days), nests are divided into those that were infected by *Rickia wasmannii* and uninfected nests. All surviving caterpillars were weighed at the start of overwintering, and the survivorship of those above (Large) and below (Small) the mean mass in infected and uninfected nests are then shown separately. Shaded regions are 95% confidence intervals around the survivorship, estimated from a proportional hazards survival model.

**Figure 5:**
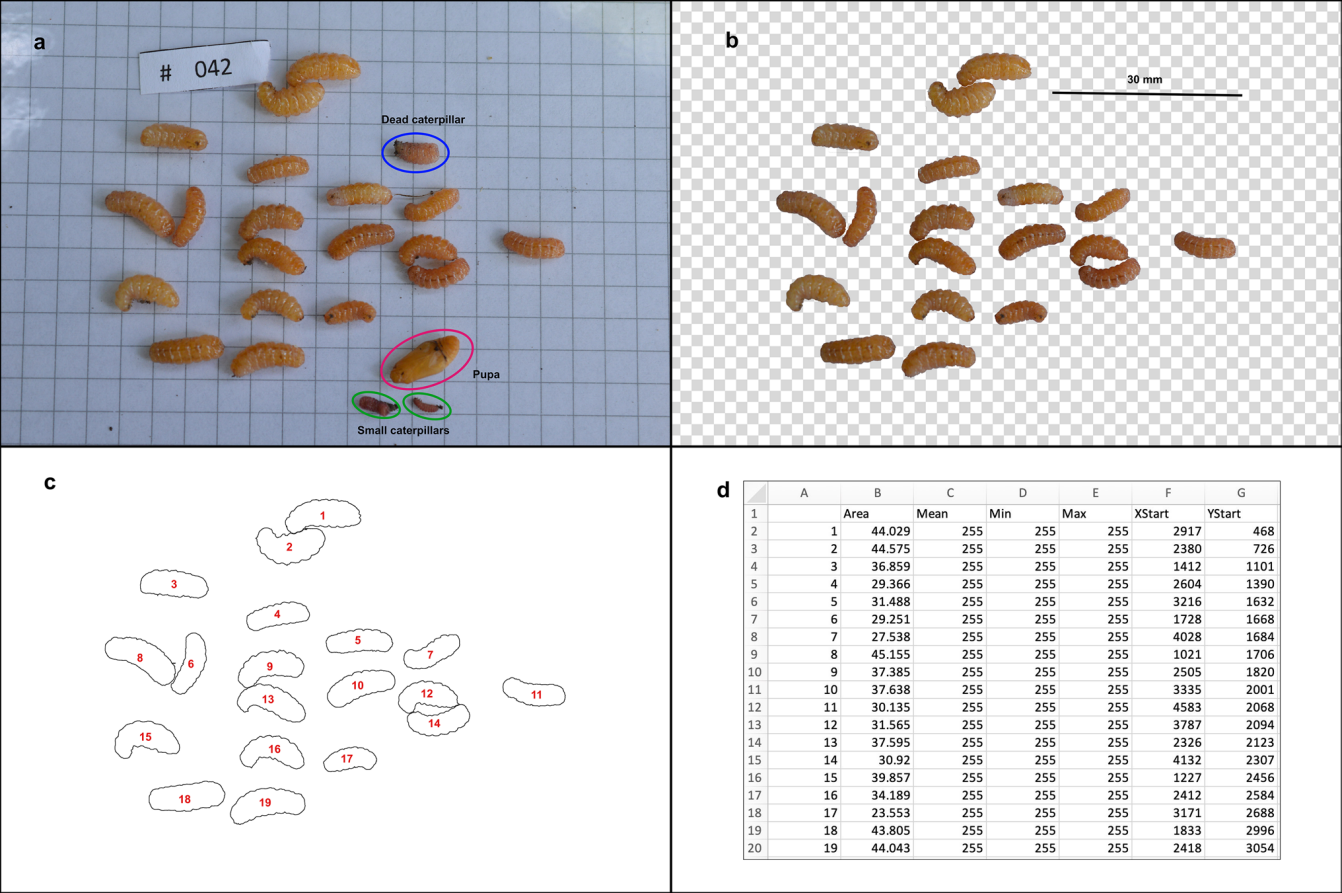
Summary of the procedure for measuring the size of prepupal *Phengaris alcon* caterpillars. (**a**) Original image taken from digital camera (by Anna Ágnes Somogyi, with 5 mm square paper background), showing 22 *P. alcon* caterpillars, including one clearly dead and two small caterpillars (which will pupate next year), and one *P. alcon* pupa. (**b**) Background and all objects other than live prepupal caterpillars removed and scale bar added (based on 5 mm squares) in Adobe Photoshop. (**c**) Tracing of perimeter of prepupal caterpillars in Fiji. (**d**) Export of details of each prepupal caterpillar from Fiji as a CSV file (here shown in Microsoft Excel), including calculated area in mm^2^.

**Figure 6:**
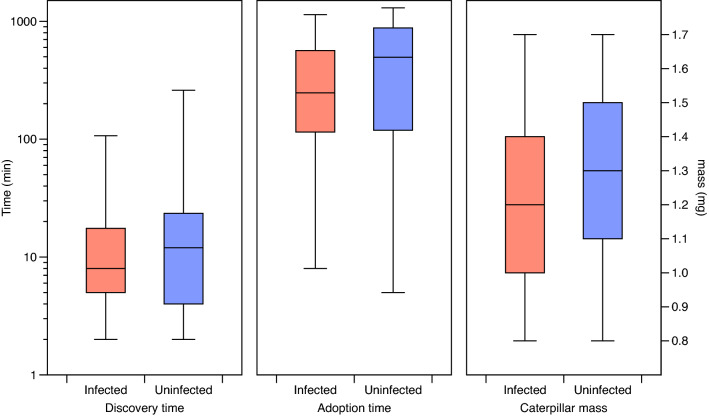
Box-plots summarising measurements of *Phengaris alcon* caterpillars before and during adoption by mini colonies of *Myrmica scabrinodis* that are either infected by *Rickia wasmannii* (red) or uninfected (blue). The whiskers show the entire range of observations.

**Figure 7:**
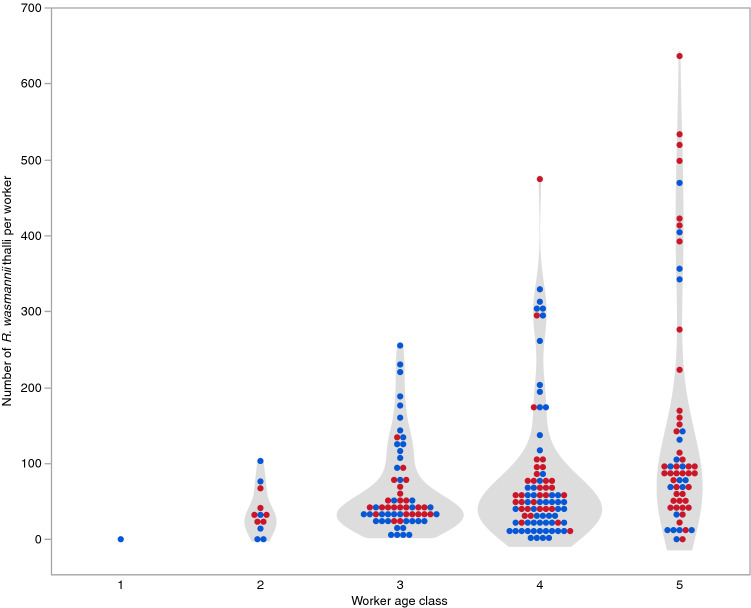
Violin plot with superimposed raw values showing the number of thalli of *Rickia wasmannii* found on workers of *Myrmica scabrinodis* of different ages. Red points represent workers sampled from colonies with *Phengaris alcon* caterpillars present, while blue points are from unparasitized colonies.

**Figure 8:**
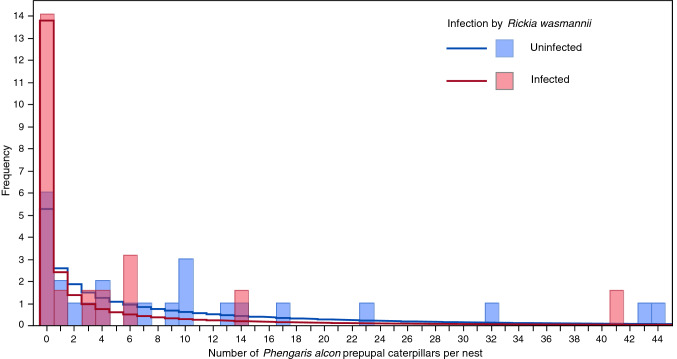
Histograms showing the distribution of number of prepupal caterpillars of *Phengaris alcon* in nests of *Myrmica scabrinodis* that are either infected by *Rickia wasmannii* (red bars) or uninfected (blue bars). Superimposed on the histogram are the fitted negative binomial probability density plots for infected (red line) and uninfected (blue line) nests.

**Figure 9:**
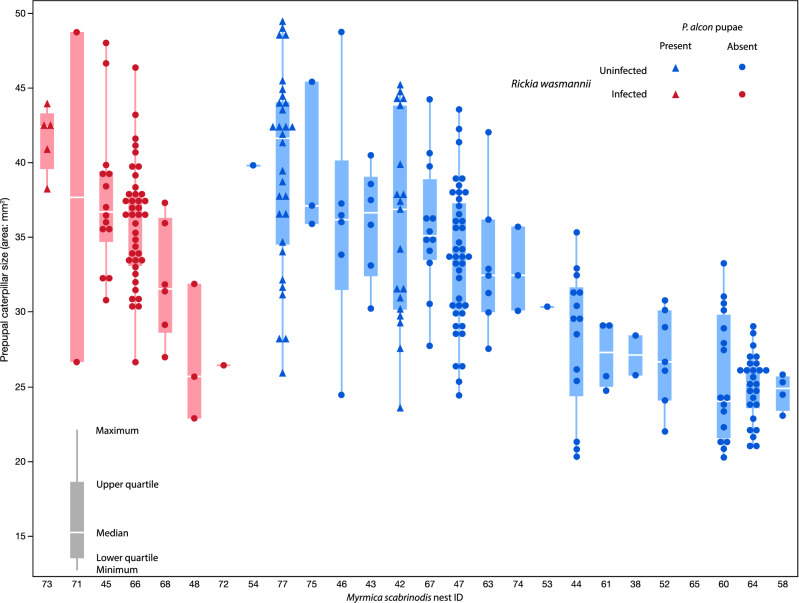
Box-plots with overlaid raw data showing the sizes of prepupal caterpillars of *Phengaris alcon* (measured as caterpillar area on field photographs) in nests of *Myrmica scabrinodis* with (red symbols) or without (blue symbols) *Rickia wasmannii* infection, and with (triangles) or without (circles) *P. alcon* pupae present. Nests are sorted along the x-axis by decreasing mean caterpillar size separately for infected and uninfected nests.

In this study we examined the effects of *R. wasmannii* on the adoption and long-term development and survival of *P. alcon*. Specifically, we investigated whether *R. wasmannii* infection of *M. scabrinodis* colonies may have an effect on any of the following traits: (1) the discovery time of *P. alcon* caterpillars; (2) the adoption time of *P. alcon* caterpillars; (3) the overwintering survival of adopted *P. alcon* caterpillars; (4) the proportion of *P. alcon* parasitized nests in the field; (5) the level of *P. alcon* parasitism in the field; and (6) the size of prepupal *P. alcon* larvae in the field (Figs. 2, 3, 4, 5, 6, 7, 8 and 9).

## Results

### Adoption and survival of caterpillars in the lab

For those caterpillars that were adopted, no significant difference between infected and uninfected colonies was observed in *discovery time* (F_1,35_ = 0.245, p = 0.623), *adoption time* (F_1,35_ = 1.28, p = 0.266), or mass of caterpillars at time of introduction (F_1,35_ = 0.497, p = 0.486; Fig. 6). These quantities also did not differ between the original colony fragments (F_11,35_ = 1.12, p = 0.374; F_11,35_ = 1.33, p = 0.249; F_11,35_ = 1.62, p = 0.135 respectively).

Of the 35 caterpillars introduced into uninfected mini *M. scabrinodis* colonies, six (17%) survived the overwintering process, but none of the caterpillars introduced into infected colonies survived this long (Fig. 4), leading to a significant difference in survival probability (Wald χ^2^ = 5.77, df = 1, p = 0.0162). There was also a significant effect of original colony fragment on survival probability (nested within *R. wasmannii* infection; Wald χ^2^ = 25.1, df = 12, p = 0.0144). For those caterpillars that were re-weighed in November, there was also a significantly higher survival for those in uninfected nests (Wald χ^2^ = 10.87, df = 1, p = 0.0010), and a positive association between mass and survival (Wald χ^2^ = 38.3, df = 1, p < 0.0001), but there was also a significant interaction between *R. wasmannii* infection and mass (Wald χ^2^ = 8.89, df = 1, p = 0.0042), with smaller caterpillars in infected nests showing disproportionately high mortality (Fig. 4). In addition, there was a significant effect of original colony fragment on survival probability (nested within *R. wasmannii* infection; Wald χ^2^ = 28.4, df = 12, p = 0.0047).

### Patterns of *P. alcon* and* R. wasmannii* infection of *M. scabrinodis* in the field

Of the 41 nests of *M. scabrinodis* found within 2 m of a *Gentiana pneumonanthe* (Dicotyledonopsida: Gentianaceae) plant, 15 (37%) were identified as infected with *R. wasmannii* in the field. When workers from these were examined under the microscope in the laboratory, the number of thalli of *R. wasmannii* found on workers was highly variable between individuals (i.e., aggregated, as is typical for macroparasites^36^). Fitting a generalized linear model with negative binomial errors to the data on number of thalli per individual from infected nests (fitted dispersion parameter *θ* = 4.39) showed a highly significant difference between nests in the level of infection (Wald χ^2^ = 245.05, df = 1, p < 0.0001), and also a strong effect of estimated worker age (Wald χ^2^ = 70.3, df = 4, p < 0.0001), with older workers having a higher load of thalli (Fig. 7), but no association with the presence or absence of *P. alcon* caterpillars (Wald χ^2^ = 0.719, df = 1, p = 0.396). Only one callow worker (age class 1) was sampled, which bore no thalli.

Twenty six of the 41 M*. scabrinodis* nests (63%) were parasitized by *P. alcon,* with between 1 and 44 caterpillars per nest. This aggregative distribution was again well-described by a negative binomial distribution, with dispersion parameter *θ* = 2.80 (Fig. 8), and there was no association between infection with *R. wasmannii* and the number of caterpillars *P. alcon* present (Wald χ^2^ = 0.1.94, df = 1, p = 0.163). Joint infection and parasitism of nests was less common than expected if both parasites acted independently (χ^2^ = 4.37, df = 1, p = 0.037).

The size (area on photographs) of *P. alcon* prepupal caterpillars in each nest varied considerably between nests (variance explained = 45%, Wald p = 0.009; Fig. 9), but was significantly larger in nests that also contained one or more pupae (F_1,19.9_ = 16.46, p = 0.025). There was no association between the total number of *P. alcon* within a nest and the size of prepupal *P. alcon* caterpillars (F_1,17_ = 0.005, p = 0.944). Caterpillars were somewhat larger in nests infected with *R. wasmannii* (Least squared mean ± SE: 39.4 ± 2.57 mm^2^) than those that were uninfected (32.1 ± 1.12 mm^2^), although not significantly so (F_1,20.5_ = 1.75, p = 0.201). It is notable that a simple analysis of caterpillar sizes without taking between-nest differences into account would have led to the conclusion that prepupal caterpillars are significantly larger in infected nests of *M. scabrinodis* (t_279_ = 4.2, p < 0.0001).

When the number of prepupal caterpillars and pupae was divided by the number of nests, this index of infection level^37^ was higher in uninfected (10.12) than in infected (4.69) nests.

## Discussion

Based on our results, it is clear that *R. wasmannii* infection of *M. scabrinodis* affects the survival and development of *P. alcon* caterpillars, which suggests competition^1,2^ between these two ant associates, although this effect is subtle.

Interestingly, there was no significant difference between infected and uninfected *M. scabrinodis* mini colonies in the discovery time of pre-adopted *P. alcon* caterpillars (Fig. 6). This is in contrast with the results of Csata et al.^14^, who showed lower discovery of *P. alcon* caterpillars within 120 min by uninfected than infected colonies, but is in harmony with the finding that *R. wasmannii* infection does not affect the locomotory behaviour of *M. scabrinodis* workers^38^. A potential reason for such a contrast is the longer time allowed in our experiment (see below), or differences in the populations studied. We also found no significant difference in adoption time between infected and uninfected mini colonies (Fig. 6). Csata et al.^14^ did not measure discovery and adoption time separately, but found no difference in the overall time between introduction and adoption between uninfected and infected colonies for those caterpillars that were adopted. It is noteworthy that in 39 out of 48 cases where caterpillars were adopted in our study (81%), it took longer than 120 min since initial introduction. However, all but one of the 70 introduced caterpillar were discovered within this period (Fig. 6).

The pattern of survival of adopted caterpillars was significantly different in uninfected and infected mini colonies, with somewhat higher survival in uninfected colonies both before and after (but not during) overwintering (Fig. 4). For those caterpillars that survived to enter the wintering phase, there was a strong effect of both infection of its host mini colonies by *R. wasmannii* and size of each caterpillar on its chance of survival, and these effects were synergistic, such that small caterpillars in infected mini colonies had a considerably lower survival probability than that predicted based on their size and the infection status of their host nests alone (Fig. 4). When we examined colonies of *M. scabrinodis* in the field, we found that the number of colonies simultaneously infected with *R. wasmannii* and parasitized by *P. alcon* was lower than expected if the two parasites were acting independently of each other. This pattern has also previously been found for co-infection of *Myrmica* colonies by different *Phengaris* species and/or *Microdon myrmicae*^24,25^.We also found that prepupal caterpillars of *P. alcon* were larger in nests where some caterpillars had already pupated, and there was a tendency for prepupal caterpillars to be larger in infected than uninfected ant nests (Fig. 9).

Putting these pieces of evidence together suggests that infection of an ant colony by *R. wasmannii* has a disproportionately large effect on the survival and development of smaller caterpillars of *P. alcon*, but rather little effect on larger caterpillars; this is what results in the somewhat larger sizes of prepupal caterpillars in infected nests. Increased mortality among small caterpillars would also lead to the lower-than-chance occurrence of co-coinfection observed in field colonies, although from our spring surveys it was not possible to demonstrate that this mortality has taken place. In general, we found evidence supporting an overall negative effect of *R. wasmannii* on *P. alcon* caterpillars, although these effects were relatively minor, and did not prevent *R. wasmannii* infected *M. scabrinodis* from raising a relatively high number of *P. alcon* in this population. Much lower numbers of *P. alcon* (and lower proportions of infected nests) are frequently found in other populations, including those where *M. scabrinodis* is the only host^25^. The only putatively positive effect of *R. wasmannii* infection on *P. alcon* was the apparent larger size of its prepupal caterpillars in infected nests compared to uninfected nests (Fig. 9), although this is likely an artefact due to the removal of smaller caterpillars from the population. However, it cannot be ruled out that the lack of competition that this could produce would increase adult fecundity or mating success^39,40^. In this regard it is relevant to note that we found no relationship between the number of *P. alcon* and size of prepupal caterpillars in this population, as might be expected if there was strong competition^41^.

It is clear that there is a large amount of between-host-colony variation in survival and growth of *P. alcon* caterpillars, and the putative effects of *R. wasmannii* on *P. alcon* (or vice versa). Some of this probably reflects the resources available to the ant colony (and hence to its parasites), and it is likely that colony size (and hence worker force and brood availability) are also important^34,42^. Nevertheless, the significant effect of original colony on survivorship of caterpillars of *P. alcon* under controlled conditions also suggests that other intrinsic properties of colonies are also important. Such a property could be the between-colony variation in cuticular hydrocarbon profiles, which has been shown to have a major effect on adoption and survival of *Phengaris* caterpillars^25,42–44^. It is known that *R. wasmannii* infection changes the cuticular hydrocarbon profile and the overall hydrocarbon abundance of *M. scabrinodis*^14^. Based on our results, however, it seems that there is unlikely to be an effect of *R. wasmannii* on adoption of *P. alcon* caterpillars, which is the stage where matching of hydrocarbons with those of the host is critical^42,45,46^, but subsequent survival and growth may be impacted by the fungus. *Phengaris alcon* caterpillars can successfully switch host species during larval development^47,48^, and this may be facilitated by the ability of larvae to change their own hydrocarbon profiles^44,49^ but may also depend on other colony characteristics^47^.

Between-colony differences in aggression and susceptibility to parasitism are also likely to be important, which may be linked to colony social structure^50^ as well as to coevolutionary arms races between parasites and hosts^42^. We still do not know whether these effects are largely genetically or environmentally determined^51–55^, but in any case the large between-colony variation suggests that large numbers of colonies need to be sampled to examine the interactions between *Myrmica* ants and their parasites^25^.

Although our results suggest an overall negative effect of *R. wasmannii* on the development of *P. alcon* caterpillars, since *P. alcon* is a rather virulent parasite of *Myrmica* nests^42,56^, while *R. wasmannii* is much less virulent^14,30–32^, the possibility exists that infection by *R. wasmannii* could be beneficial for *M. scabrinodis* nests if *P. alcon* is common in a population. The reported negative effects of *R. wasmannii* on its *Myrmica* host are also mostly found in laboratory studies, and their importance under field conditions is unclear. The seasonal differences in *R. wasmannii* infection^57^ could be considered when laboratory experiments are translated to field conditions. While increased allo- and auto-grooming frequency^30^, the smaller worker size^29^, and the less aggressive and more timid behaviour^14,31^ associated with *R. wasmannii* infection may have an effect on resource acquisition and territorial disputes in the field, it is difficult to imagine that these will have as large an effect on fitness as the direct consumption of brood or diversion of resources from sexual progeny by *P. alcon*. Increased water-loss and earlier mortality of *R. wasmannii* infected *M. scabrinodis* workers^32^ is also unlikely to be a major problem in the field, where *M. scabrinodis* is typically found in marshy meadows where humidity is high and water abundant.

In general, our study supports the idea that competition between parasites for host resources may have complex outcomes for both parasites and hosts^1,2^. The immediate resources used by the two parasites are different, but both draw on the overall resources of the ant colony. Our study was not designed to examine host fitness, and so it is unclear whether the combined effects of the two parasites were additive, synergistic, or antagonistic. Further experiments focussing on host fitness would be necessary to examine this aspect of the interactions. Theoretical models^1,2,7^, greenhouse and laboratory experiments^3,5^, and examples from biological control^4,6^ suggest that synergistic or additive virulence effects are most likely^58,59^, so the possibility of a conditional^60^ antagonistic effect, as outlined above, is intriguing. Such effects are likely to be more common than hitherto documented^61^; for example, such a conditional effect has recently been proposed for a microsporidian parasite of *Daphnia*^62^. There is no evidence for direct interaction between the two parasites in our study. Despite *R. wasmannii* being able to also infect myrmecophilous arthropods in *Myrmica* nests^63^, we have never observed infection of *P. alcon* by *R. wasmannii*, either in the course of this study, or when examining numerous other *P. alcon* caterpillars in Hungary and Denmark. Hence, contest (interference) competition is unlikely to be the major form of competition between the two parasites, whereas all the indirect evidence we have points to scramble (exploitation) competition for host resources, which contrasts with the intraspecific contest competition that has been demonstrated for *P. alcon*^41^. In other parasitic systems it has been argued that the type of interaction between parasites should be reflected in the association in infection levels between parasites within a host population^2^. Recent comparative studies have found more positive than negative associations between multi-parasite infections in hosts^58,59^, which has been interpreted as facilitation of infection of one parasite by the presence of another e.g.^64^. It has been suggested that such interactions are mostly amensal—where one parasite has a positive effect on the other, but there is no measurable reciprocal effect^2,65,66^. Whether this represents contest or scramble competition has been interpreted differently by different authors, but in this study we found a negative association between the two parasites, but also clear evidence of an asymmetry in their effects on each other, with *P. alcon* apparently being strongly affected by *R. wasmannii*, but not vice versa.

## Methods

### Terminology

To allow easier distinction between the two types of *Myrmica* parasites used in this study, we refer to *M. scabrinodis* nests containing *P. alcon* caterpillars as “*parasitized*”, while *M. scabrinodis* colonies and workers with *R. wasmannii* are termed “*infected*”. Similarly, “*unparasitized*” is used about nests without *P. alcon* and “*uninfected*” about nests without *R. wasmannii*. The nests containing neither *R. wasmannii* nor *P. alcon* are referred to as “*healthy*”. “*Colony fragments*” from wild *M. scabrinodis* nests were collected to give workers for the “*mini colonies*” for the lab work (see details: Adoption and survival of caterpillars in the lab). “*Larvae*” is used to refer to ant larvae, while the larvae of the butterflies are called “*caterpillars*”. We use the term “*social parasite*” to refer to any parasite that lives on the resources of a social insect colony rather than the individual members of that colony^67^ rather than in the narrow sense of an ant species that exploits another ant species^19^.

### Study species, population and site

The sample site was in northern Hungary at Gyöngyös: Sár-hegy: Gyilkos-rét (47°48' N, 19°58' E; 352 m a.s.l). It is a small (ca. 0.4 ha), marshy meadow with tall-sedge and dense stands of *Gentiana pneumonanthe*, the initial foodplant of *P. alcon*, surrounded by oak forest (Fig. 2).

We sampled nests of *M. scabrinodis* (Fig. 1, Supplementary Video S1), which is a common ant species on this meadow that is, as far as is known, the only host of *P. alcon* and *R. wasmannii* at this site^25,68^. Numerous healthy, infected, parasitized or co-infected *M. scabrinodis* nests can be found at Gyilkos-rét^25^, making this site ideal for this study. To confirm the identity of putative *M. scabrinodis* nests, 5–10 workers from each nests were examined under a 40 × magnifying hand lens in the field, and then transferred to vials of 67.5% ethanol to confirm identification in the lab by AT, using a Leica MZ125 microscope (Wetzlar, Germany) at 10–160 × magnification and keys by Radchenko and Elmes^69^.

*Rickia wasmannii* (Fig. 1, Supplementary Video S1) is one of the most widespread ectoparasitic^70^ Laboulbeniales fungal species in Europe, infecting ten *Myrmica* species and some of their arthropod associates from Turkey to Portugal^63,71–73^. Several effects of Laboulbeniales fungi on their hosts are known, which are primarily negative^3,14,29–32,74^ but can also be indirectly positive^75^. Research in recent years has made *R. wasmannii* one of the best known ant-parasitic Laboulbeniales species. The presence of *R. wasmannii* on worker ants was checked along with the identification of the ants (see above) in the field by AT, and later confirmed by FB in the laboratory based on thallus morphology^76,77^.

While the Alcon blue butterfly, *P. alcon* (Fig. 1, Supplementary Video S1) is currently considered as “least concern” in the latest edition of the European red list of butterflies^78^, it is classified as “near-threatened” in Hungary^79^, and there is concern that its numbers have decreased rapidly in Europe since the last assessment^80^. Its caterpillars start their development feeding on seeds of different *Gentiana* host plant species. In the final instar phase, the caterpillars leave the initial food plants and mimic the odour^42–44^ and the sound^81^ of certain *Myrmica* species, so as to be “adopted” and raised by the ants^82^. These ‘cuckoo’ caterpillars are mostly fed by host workers with trophallactic regurgitations but can also feed directly on ant brood^26^. The abundant *P. alcon* population at Gyilkos-rét belongs to the *G. pneumonanthe*-using, hygrophilic form (*P. alcon* H)^25^. This population typically flies in July and the caterpillars leave the host plants for adoption in August, depending on annual weather conditions (AT, pers. obs.).

### Adoption and survival of caterpillars in the lab

Because *M. scabrinodis* is a polygynous species^69^, it is easy to collect colony fragments with a few (but not all) queens, hundreds of workers, and brood without extirpating the “mother” colonies. We collected 7 infected and 7 uninfected colony fragments on the 4th August. Ants were kept in plastic boxes (16.5 cm × 11.5 cm × 6 cm) treated with Fluon (ICI, London, GB) on their inner walls to prevent them from escaping. Inside these boxes, we created nest chambers (5.5 cm × 4.5 cm × 1 cm) with plaster floors, covered with glass plates. The ants were kept at room temperature (22 ± 1 °C) under a natural light cycle, and fed with frozen cockroaches on Thursdays and with 20–20% honey–sugar water solution on Mondays and Thursdays prior to wintering. Water was available ad libitum.

Five small artificial “mini” *M. scabrinodis* colonies were derived from each of the 7 infected and 7 uninfected colony fragments and used for the tests, resulting in 35 infected and 35 uninfected mini colonies in total. Each mini colony was set up without either queen or brood, and contained 20 workers. They were kept under the same conditions as their mother colonies, except that their plastic boxes were round (60 mm diam.), and the nest chambers were smaller (2 cm × 1.5 cm × 1 cm; Fig. 3). If any of the 20 workers died during the course of the experiment, they were replaced by others from their mother colony fragment.

For wintering (12 November–24 April, see below for details), the nests were moved to a climate-controlled chamber where the temperature was gradually decreased to 7 ± 2 °C, and then increased back to 22 ± 1 °C at the end of winter (Fig. 4).

To obtain pre-adopted *P. alcon* caterpillars, 15 stems of *G. pneumonanthe* bearing eggs of *P. alcon* were collected from strong plants in the field, and moved to the lab on 4 August. In the lab, stems were kept in a glass of water placed in a plastic basin. They were kept fresh for 2–3 weeks while the caterpillars emerged. One young fourth instar *P. alcon* caterpillar, freshly dropped from their *G. pneumonanthe* initial host plants, was weighed to the nearest 0.1 mg using an OHAUS Pioneer PA64C analytical balance (Parsippany, NJ) and introduced to each mini colony using a fine brush. The time spent between introduction and the first contact with a worker (*discovery time*) as well as the time between the first contact and adoption (*adoption time*) were recorded. The survival of the caterpillars was monitored weekly (on Thursdays), except during wintering, when they were disturbed less frequently. Surviving caterpillars were weighed again before the start of the wintering period, on 12 November (week 14). This study was run until the 24 April (week 34), when only one caterpillar was still alive (in an uninfected mini colony; Fig. 4).

### Patterns of *P. alcon* and* R. wasmannii* infection of *M. scabrinodis* in the field

*Myrmica scabrinodis* nests within 2 m of *G. pneumonanthe* plants (the approximate foraging zone of *Myrmica* workers^83^) were located on 23 June. The nests were carefully opened and searched for fully-grown *P. alcon* larvae and pupae. The number of larvae and pupae were noted and if any were present, photographs taken in the field in order to measure their size (Fig. 5). At least 15 workers from each colony were collected and transferred to the laboratory in vials of 67.5% ethanol. Host ant identity was confirmed and workers were examined for presence of *R. wasmannii* under a Leica MZ125 microscope at 10–160 × magnification. Any *P. alcon* were then carefully replaced in the field nest, and the nesting material restored as far as possible.

### Measuring prepupal caterpillars

The collected *P. alcon* caterpillars (and pupae) from each nest were placed onto a sheet of 5-mm square paper, and organized such that they were well separated (Fig. 5, Supplementary Note S2). Photographs of caterpillars from each nest were taken using a Nikon D3200 camera (Tokyo, Japan) with Nikon AF-S Micro NIKKOR 40 mm 1:2.8 lens, hand-held directly above the caterpillars. Photographs were taken in the shade using auto-focus in shutter-priority mode, with a fixed shutter speed of 1/60 s and ISO of 400, which resulted in apertures of between *f-*3.3 and *f*-8 under field conditions. The subjectively sharpest image from each nest was used for subsequent estimation of caterpillar sizes.

The sizes of the fully developed (prepupal) caterpillars in each nest were estimated using the Fiji image processing software^84^. Since they were relatively rare, the sizes of pupae (n = 6) and small (2-year developing) caterpillars (n = 8) were not estimated, and neither were sizes of damaged or clearly dead caterpillars (n = 10). Before measuring, the photographs were edited using Adobe Photoshop 2020 (San Jose, CA) to remove small traces of soil, seeds and weeds, and to create a scale bar based on the graph-paper background. The edited images were opened in Fiji^84^, where the area of each caterpillar was calculated based on the scale bar, using a macro script (Supplementary plugin S3). The results were saved in CSV files for statistical analysis. See Supplementary Note S2 for more details of this process.

### Determination of age of workers and *R. wasmannii* thallus load

Altogether 225 M*. scabrinodis* workers (collected randomly from the centre of the nest) from colonies recorded as infected in the field (15 from each colony) were screened for thalli of *R. wasmannii* using a Leica MZ125 microscope at 10–160 × magnification. All fungal thalli were counted on the whole ant body. All workers collected from colonies (15 from each colony) recorded as uninfected in the field were also screened, but no thalli were found. According to the coloration of the cuticle, each worker ant from infected colonies was assigned to one of five age categories following Cammaerts-Tricot^85^.

### Statistical analysis

The mass, *discovery time*, and *adoption time* of caterpillars were compared between infected and uninfected mini colonies using linear mixed models (LMMs), with original colony treated as random variable. The time variables were right-skewed and log-transformed for this analysis. Survival of caterpillars introduced into infected and uninfected colonies was compared using a proportional hazards survival model, with original colony nested within presence or absence of *R. wasmannii*. An additional survival analysis was carried out for those caterpillars that had survived until wintering and had hence been weighed on 12 November (week 14). Here caterpillar mass was also included as a covariate, together with its interaction with *R. wasmannii* infection status, and once again original colony nested within presence or absence of *R. wasmannii*.

The number of *P. alcon* caterpillars was compared between infected and uninfected nests in the field using a generalized linear model with negative binomial errors. The number of thalli of *R. wasmannii* on workers was compared between parasitized and unparasitized nests of *M. scabrinodis* using a generalized linear mixed model (GLMM) with negative binomial errors, which also included worker age as a covariate and colony as a random factor. The independence of infection by *R. wasmannii* and parasitism by *P. alcon* in the field was tested using a Chi-squared test for a 2 × 2 contingency table.

The size of prepupal caterpillars of *P. alcon* in infected and uninfected colonies was compared using a LMM, which also included a dummy variable coding whether pupae were present in the colony, and the total number of *P. alcon* (larvae and pupae) present, with colony as a random factor.

All analyses were carried out using JMP Pro v. 15.2.1 (SAS corporation, Cary, NC), except GLMMs, which were carried out using the glmer.nb() function of the R package *lme4* version 1.1-26^86^.

## Supplementary Information

Supplementary Video S1.

Supplementary note S2.

Supplementary plugin S3.

Supplementary data S4.

## Data Availability

The data generated and analyzed during this study are available for download as Supplementary data S4.
