# Supplementary material for: Ectoparasitic fungi of Myrmica ants alter the success of parasitic butterflies
Source: Sci Rep. 2021 Dec 15;11:24031. doi: 10.1038/s41598-021-02800-3 (PMC8674344; doi:10.1038/s41598-021-02800-3)
Supplement: Supplementary file 2 — Supplementary note S2. [file 41598_2021_2800_MOESM2_ESM.pdf]

How to install *Caterpillar Measurement* script in Fiji

Open Fiji, from the *Plugins* menu select *Install...*

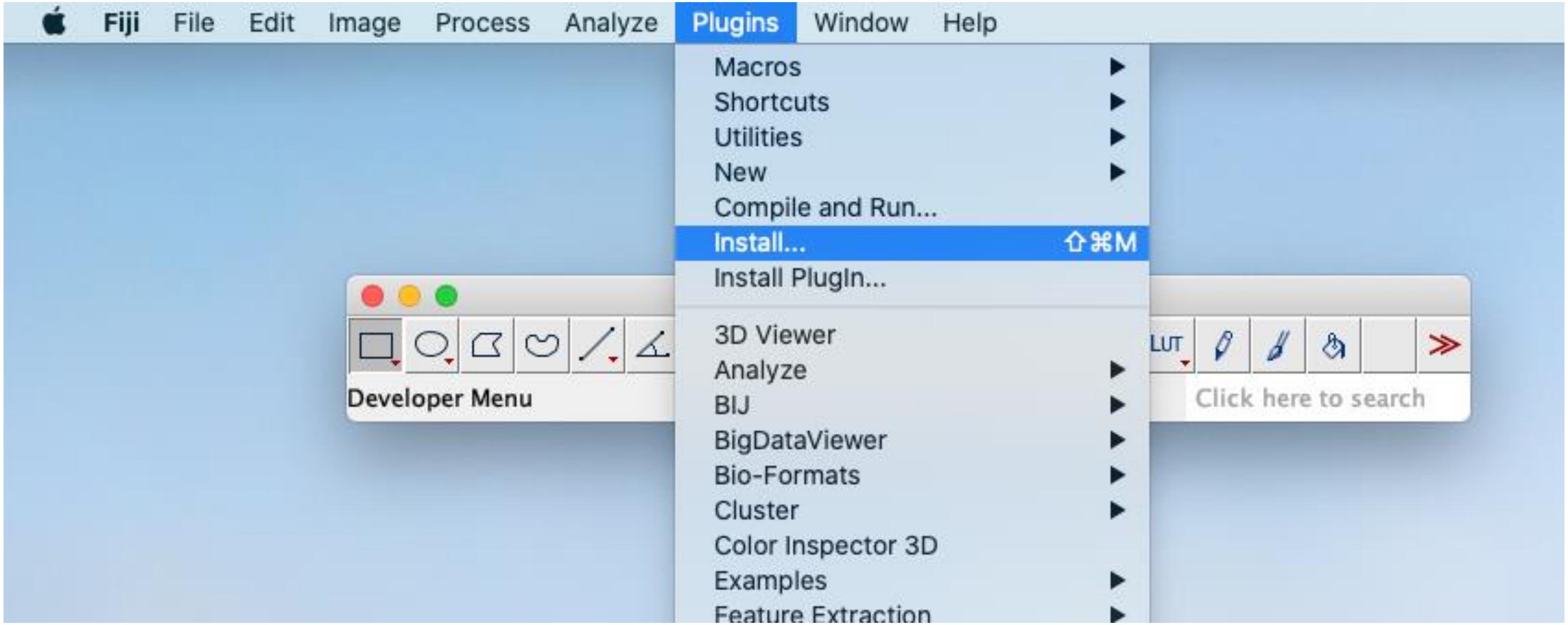

Select the *Caterpillar_Measerument.ijm* file

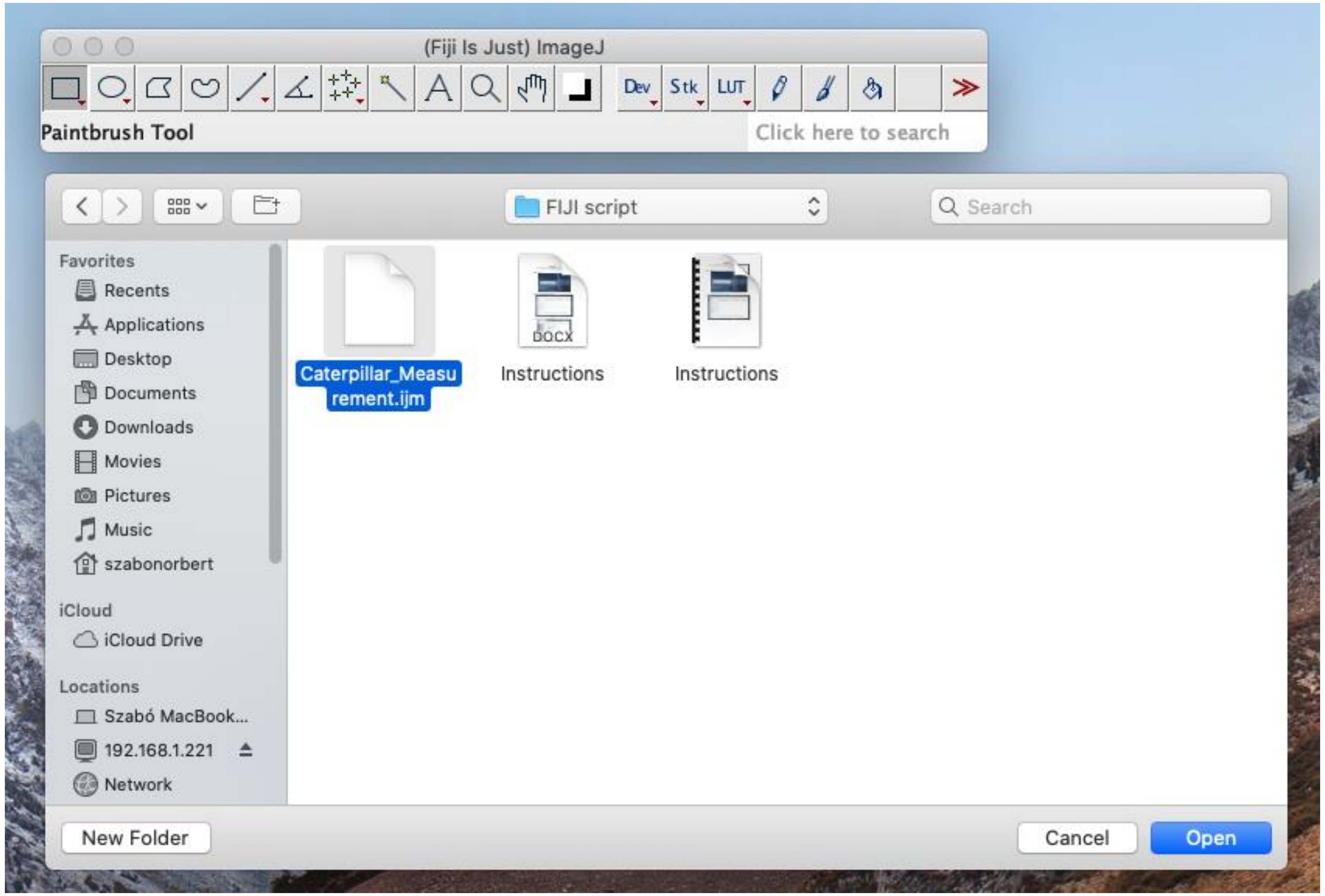

Save the script into the Fiji's *plugins* directory.

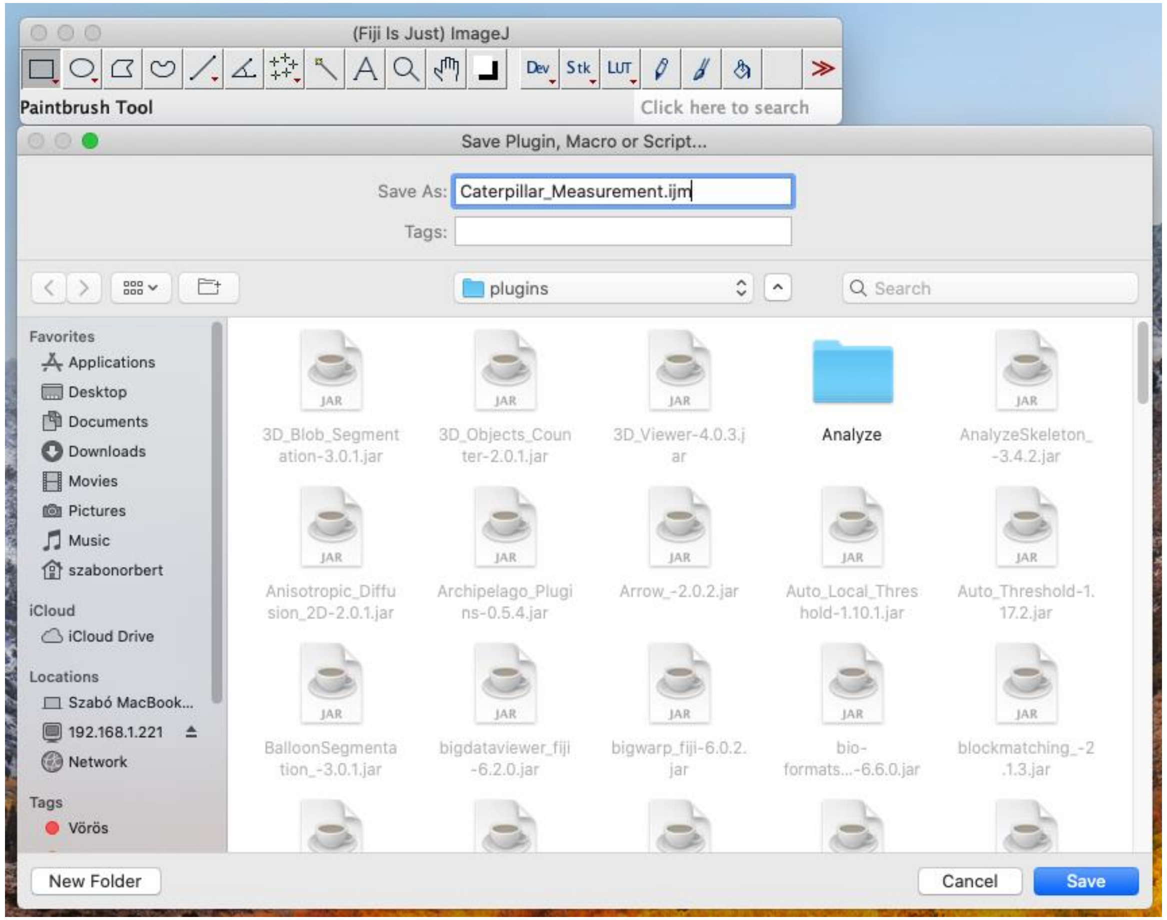

Please restart the Fiji

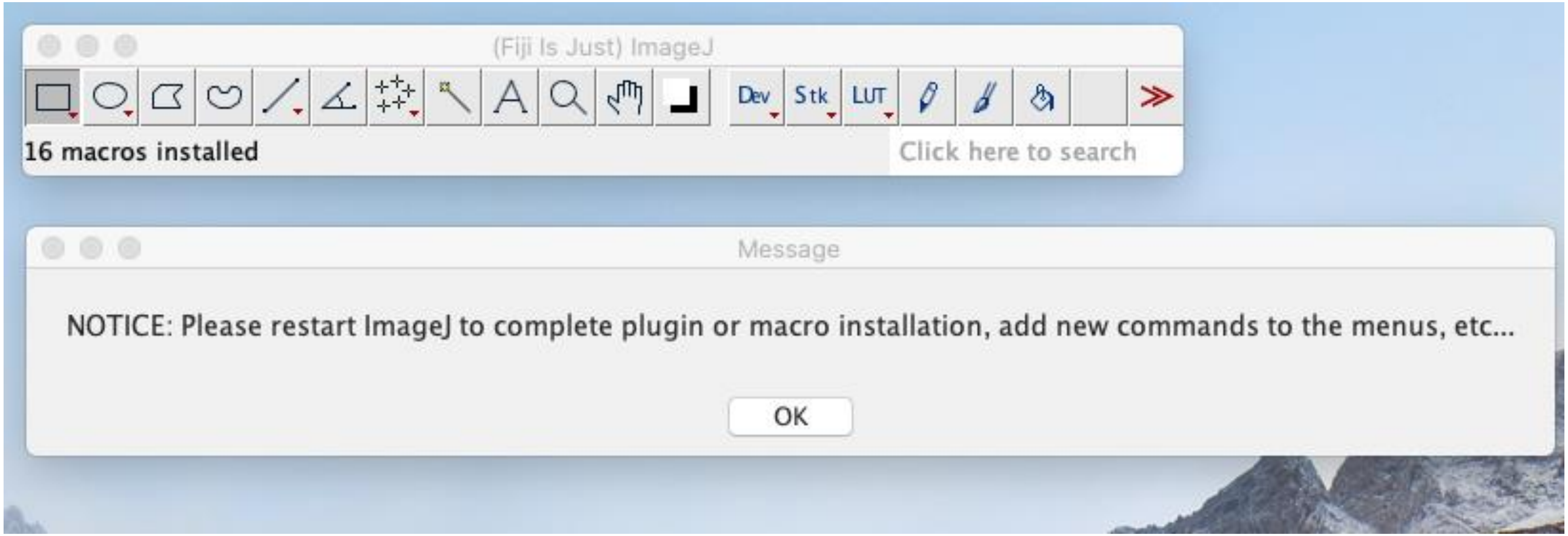

Add a new shortcut

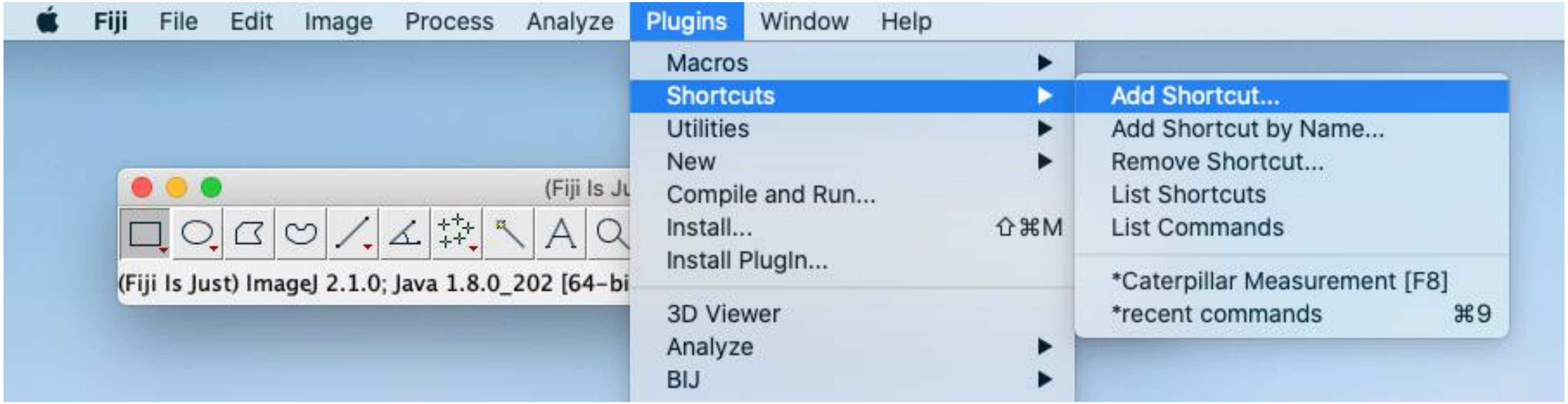

Add a new shortcut, then ready to use

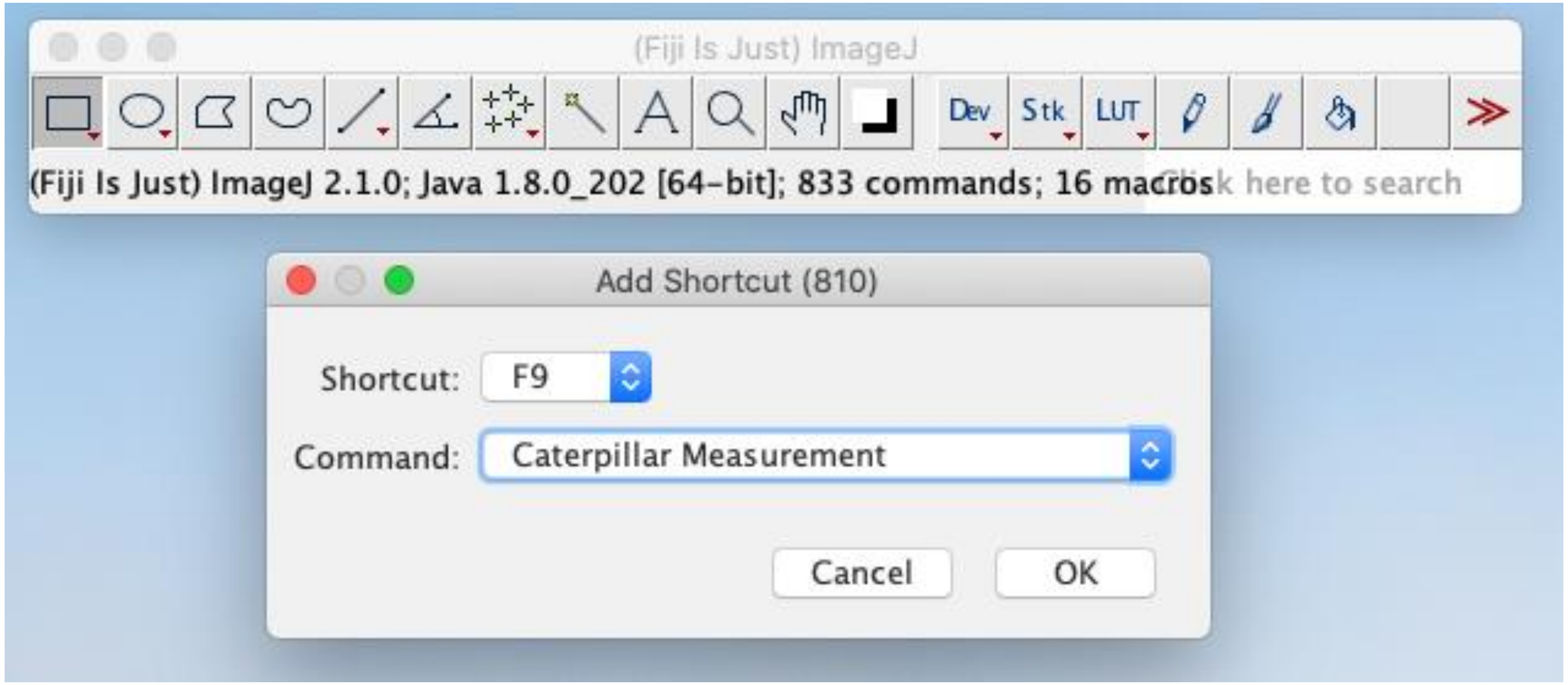

How to use Adobe Photoshop 2020 and Fiji to caterpillar measurement

Step-by-step guide

Open the image in Adobe Photoshop 2020

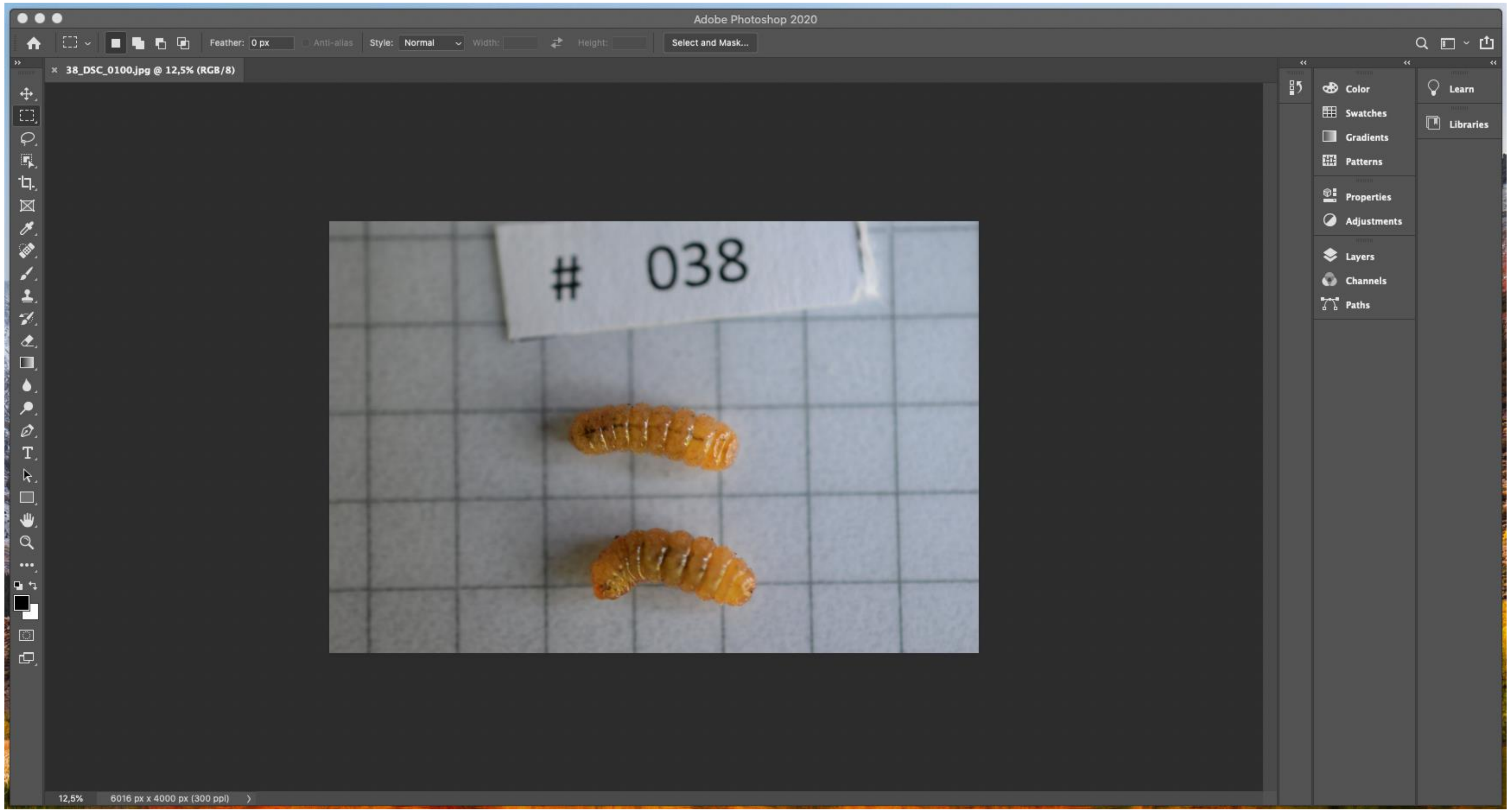

With the usage of *Quick Selection Tool* select the caterpillars

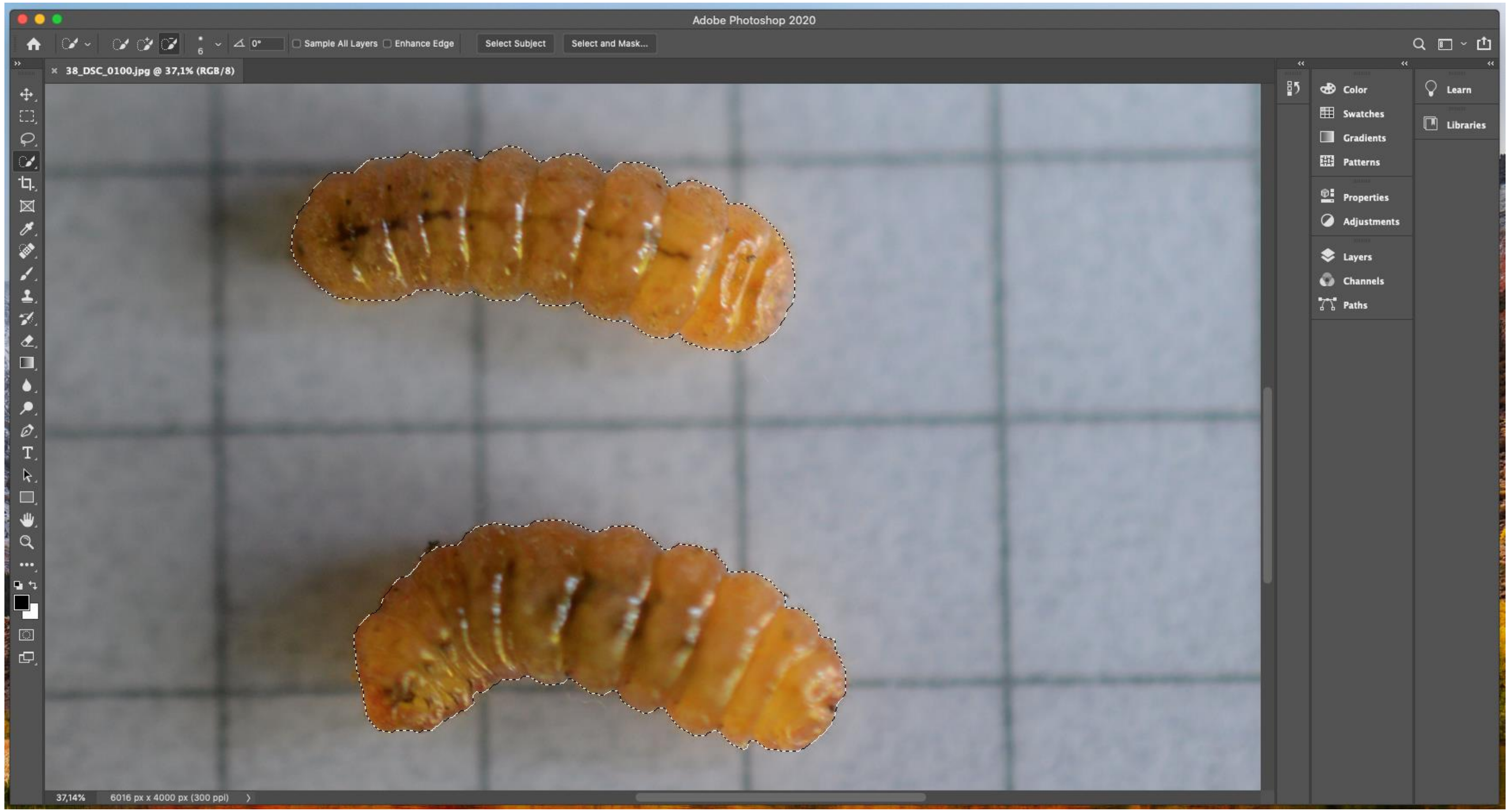

Copy the selection into a new layer

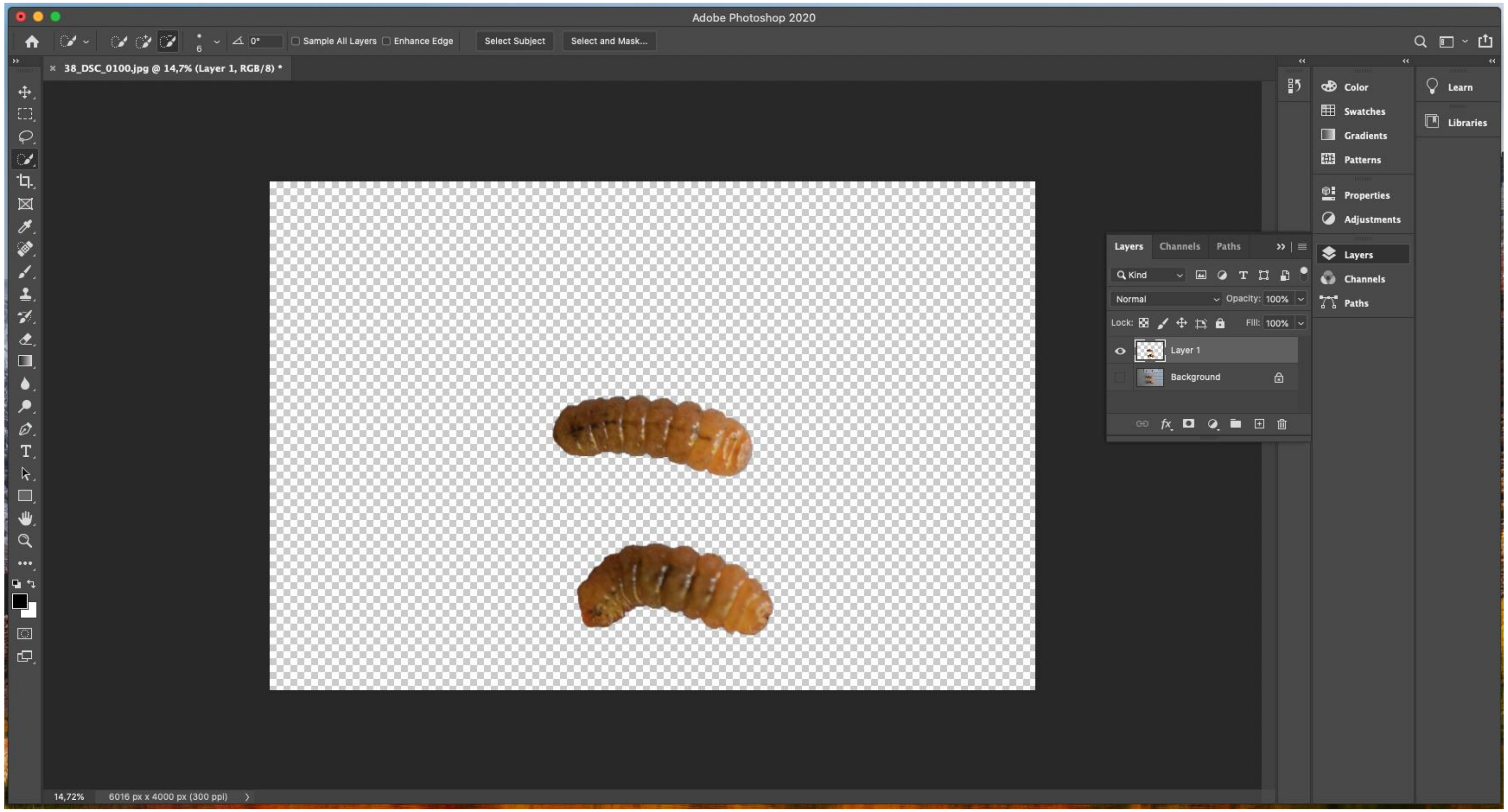

Set up a thin sizing line

(in our case it is 6 unit long)

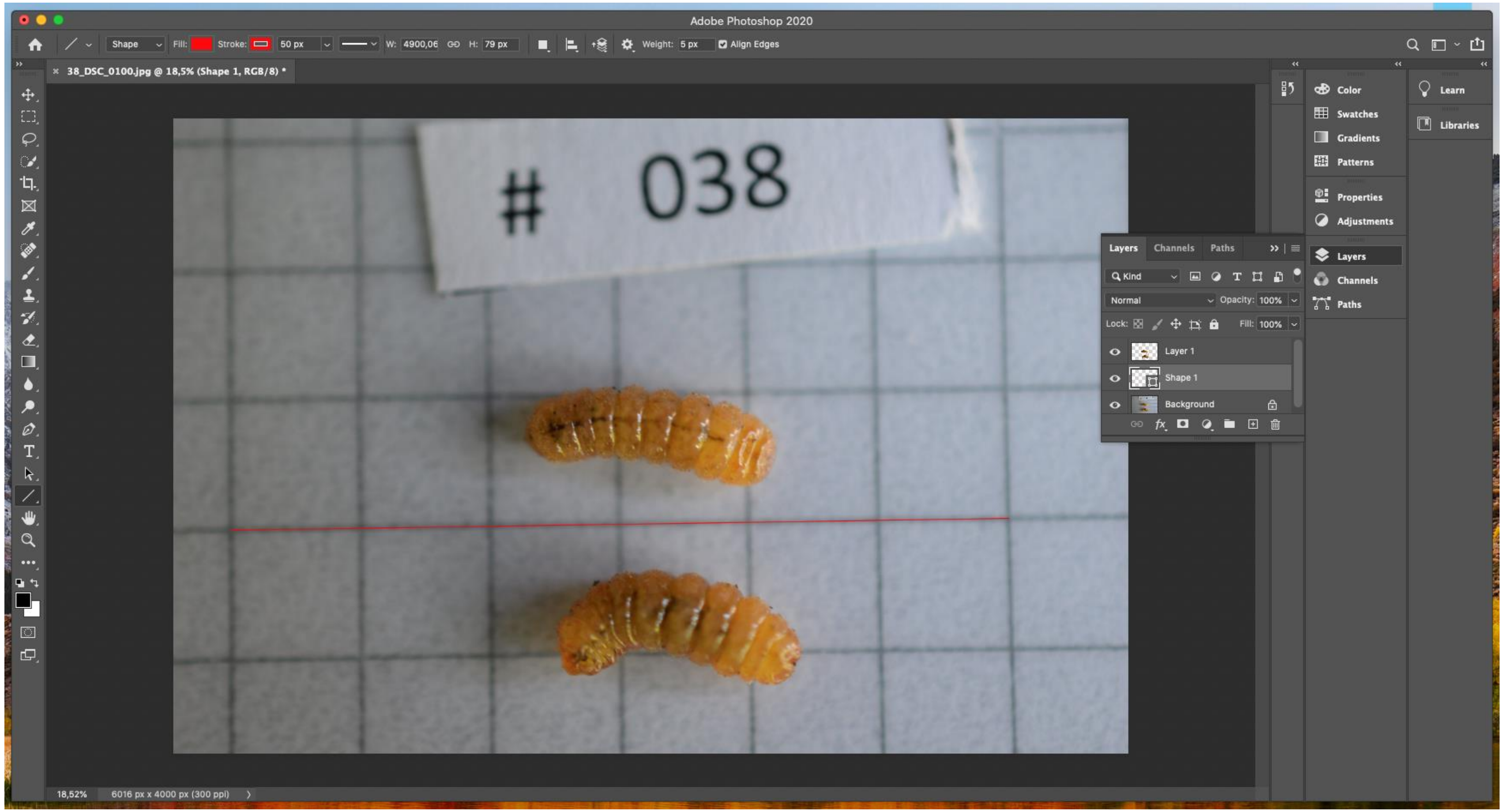

Turn off the background layer and save the image into PNG file

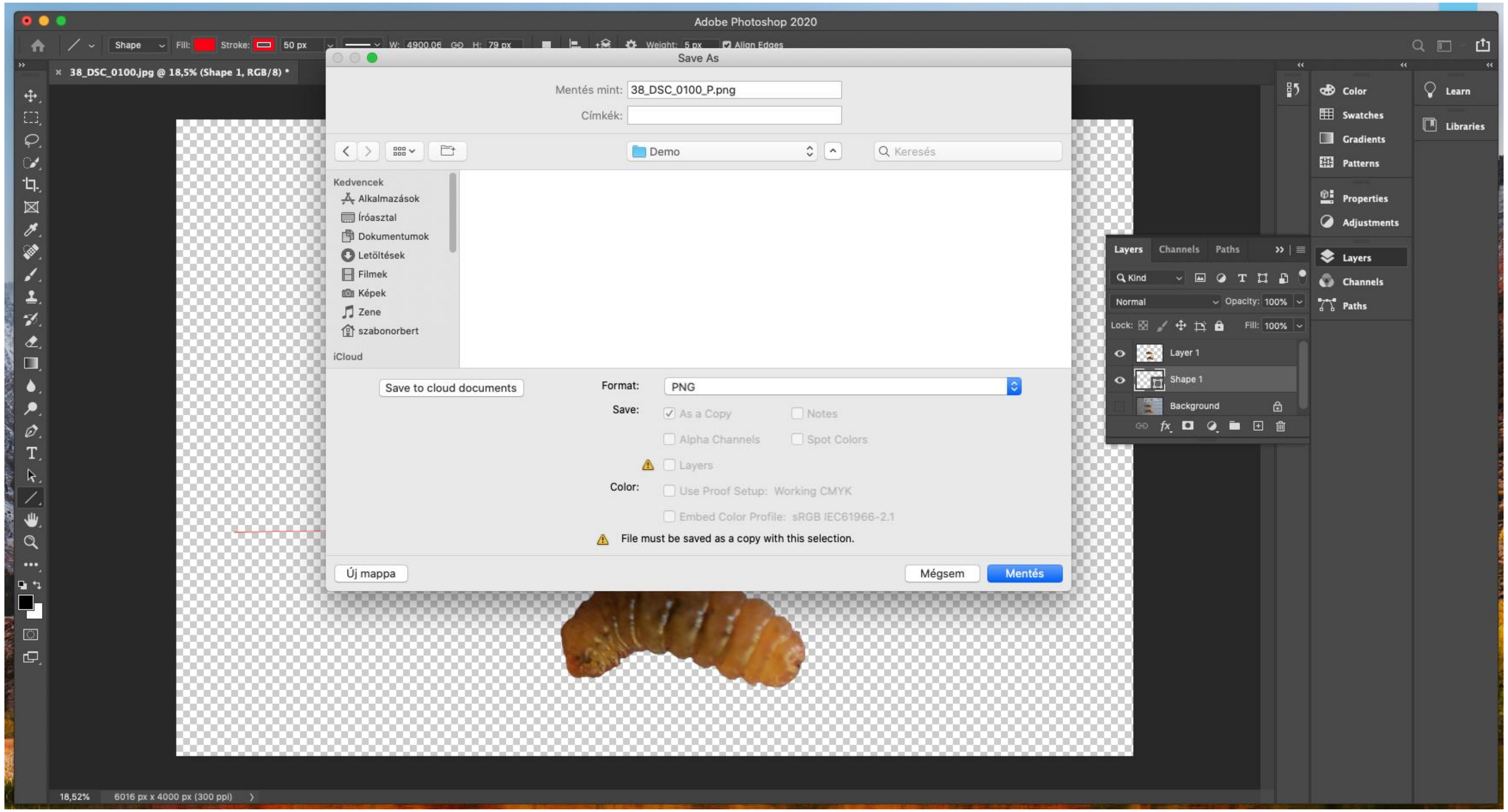

Open a preprocessed image in Fiji and set up the scaling in the Analyze → Set Scale… menu

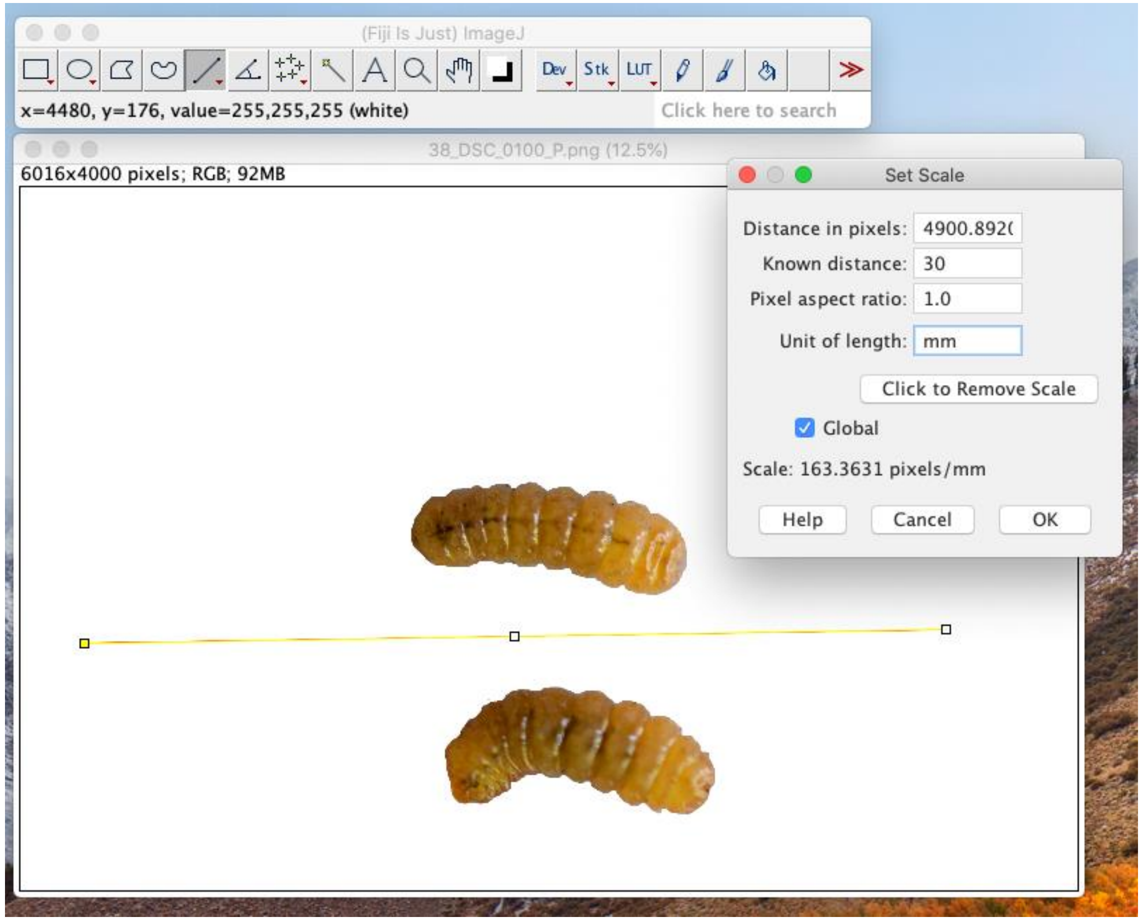

Run the *Caterpillar Measurement* macro script and check the results

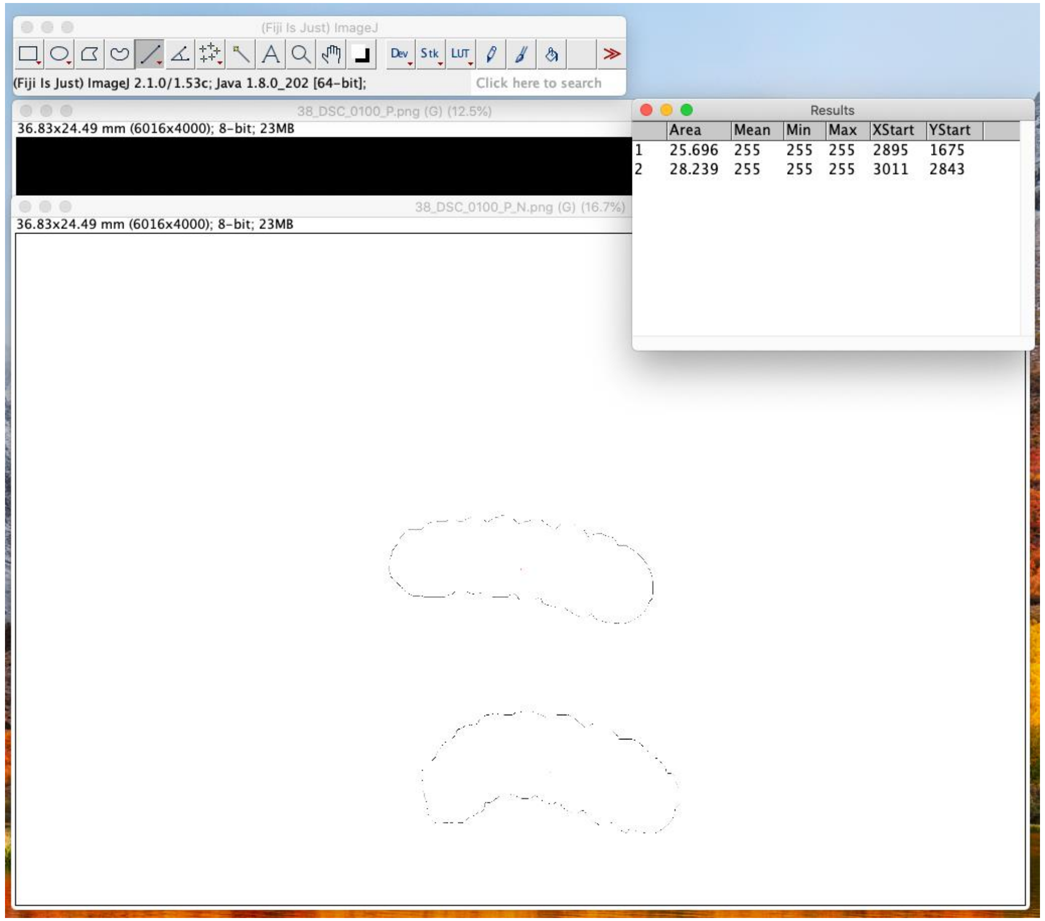

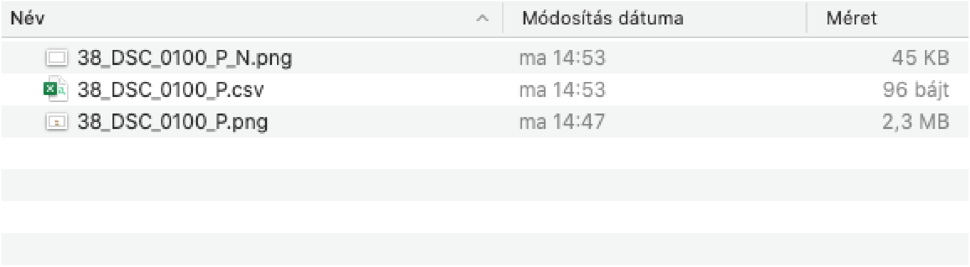

Név	Módosítás dátuma	Méret
38_DSC_0100_P_N.png	ma 14:53	45 KB
38_DSC_0100_P.csv	ma 14:53	96 bájt
38_DSC_0100_P.png	ma 14:47	2,3 MB
